# Supplementary material for: Parallel factor analysis for multidimensional decomposition of functional near-infrared spectroscopy data
Source: Neurophotonics. 2022 Nov 15;9(4):045004. doi: 10.1117/1.NPh.9.4.045004 (PMC9665873; doi:10.1117/1.NPh.9.4.045004)
Supplement: Supplementary file 1 [file NPh_009_045004_SD001.pdf]

Supplemental Material

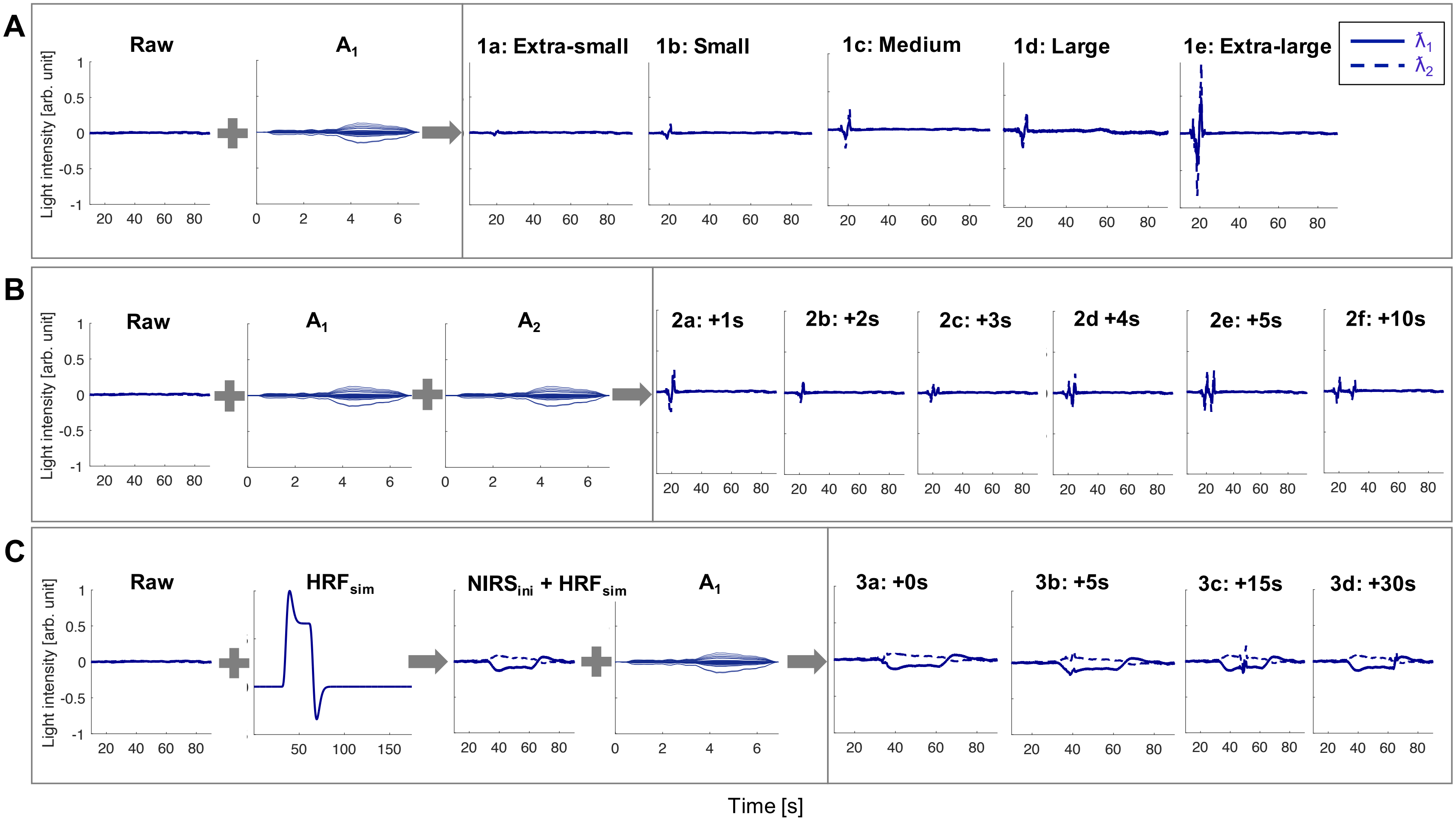

Fig. S 1 Parameters of all simulations. One or two real motion artifact(s) ($A_{1/2}$) and a synthesized HRF (HRF_{sim}) were added to a normalized resting-state fNIRS signal (Raw). A shows simulations 1a-e with an artifact of varying amplitude sizes, B simulations 2a-f with a complex artifact where the onset of the second artifact (A_2) was varied relative to the onset of the first artifact (A_1), and C simulations 3a-d with varying onset of the artifact relative to the beginning of the HRF_{sim}. $\lambda 1|2 = 690|830$ nm.

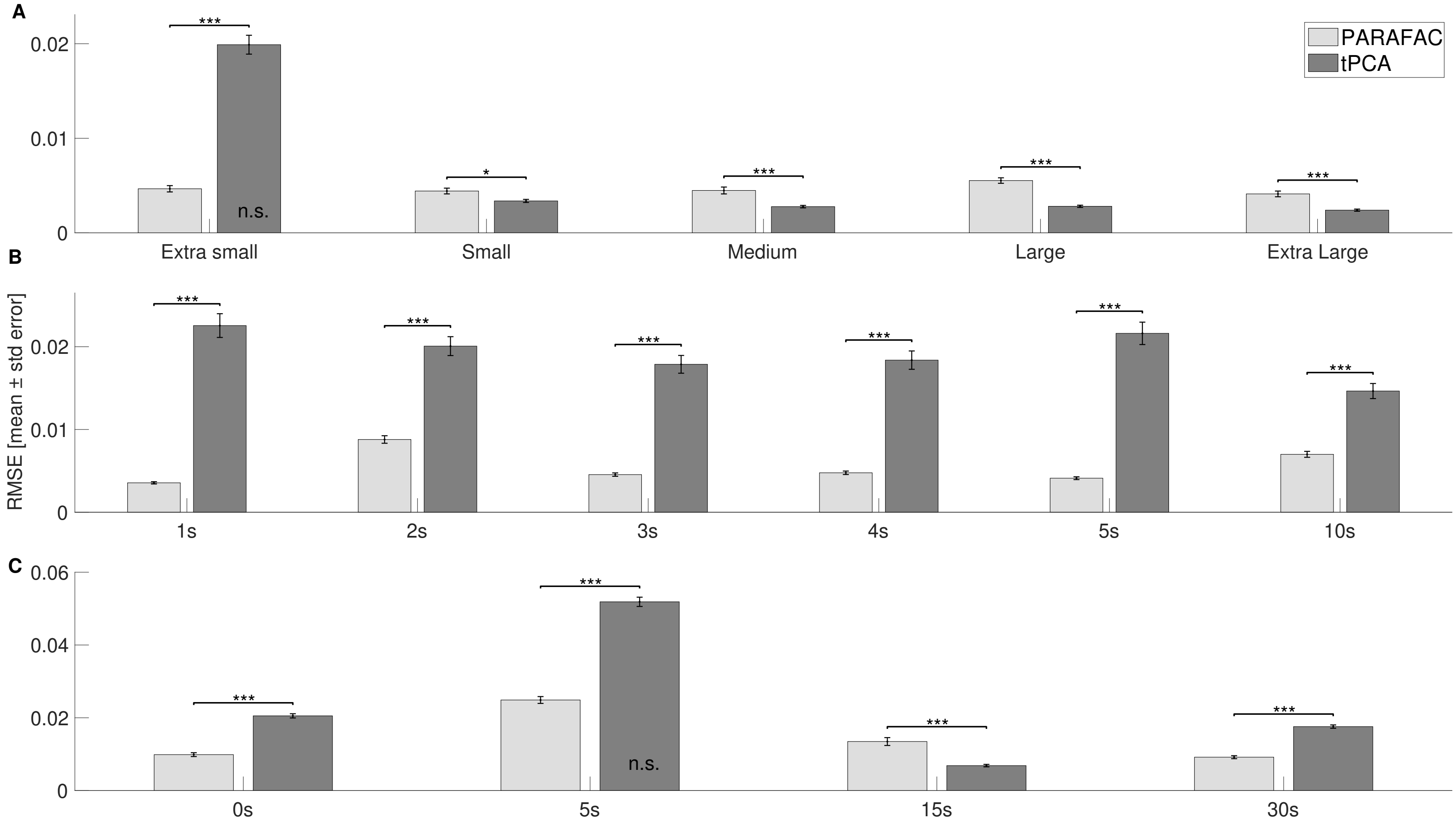

Fig. S 2 Evaluation of correction in simulated motion artifacts based on signal similarity by the use of the root mean square error (RMSE). Simulations are identified on the x-axis. A: 1a-e) artifacts with different amplitude sizes; B: 2a-f): complex artifacts with two superimposed artifacts and an onset delay between the 1st (A_1) and 2nd artifact (A_2); and C: 3a-d) the onset of the artifact relative to the beginning of a simulated HRF. A lower RMSE represents higher resemblance between the corrected and the initial clean fNIRS signal, hence a better correction of the artifact. Results are displayed separately for the correction with PARAFAC (light grey bars) and tPCA (grey bars). The RMSE of the uncorrected signal is not displayed in this figure but differed significantly from both correction techniques in all conditions, except where specified otherwise inside the bar. Significance level are based on post-hoc tests with Tukey correction. $*p \leq 0.05$, $***p \leq 0.001$, n.s. $p > 0.05$. Uncorrected = NIRS_{ini} + artifact (A_1) without correction, PARAFAC = NIRS_{ini} + artifact ($A_{1/2}$) after artifact correction with PARAFAC, tPCA = NIRS_{ini} + artifact ($A_{1/2}$) after artifact correction with tPCA.

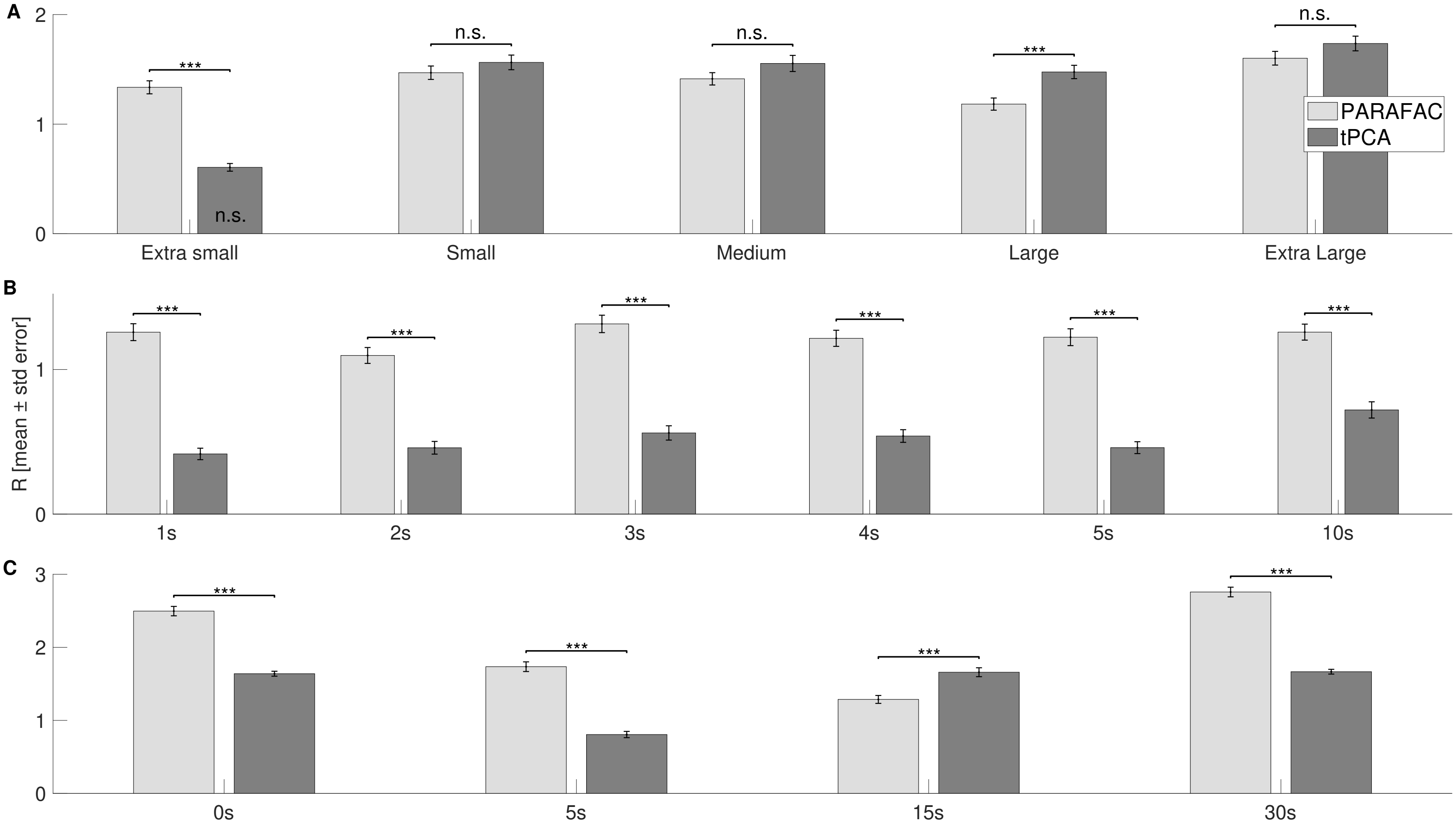

Fig. S 3 Evaluation of correction in simulated motion artifacts based on signal similarity by the use of the Pearson product-moment correlation coefficient (R). Simulations are identified on the x-axis. A: 1a-e) artifacts with different amplitude sizes; B: 2a-f): complex artifacts with two superimposed artifacts and an onset delay between the 1st (A_1) and 2nd artifact (A_2); and C: 3a-d) the onset of the artifact relative to the beginning of a simulated HRF. A higher R represents higher resemblance between the corrected and the initial clean fNIRS signal, hence a better correction of the artifact. Results are displayed separately for the correction with PARAFAC (light grey bars) and tPCA (grey bars). The R of the uncorrected signal is not displayed in this figure but differed significantly from both correction techniques in all conditions, except where specified otherwise inside the bar. Significance level are based on post-hoc tests with Tukey correction. $*p \leq 0.05$, $***p \leq 0.001$, n.s. $p > 0.05$. Uncorrected = NIRS_{ini} + artifact (A_1) without correction, PARAFAC = NIRS_{ini} + artifact ($A_{1/2}$) after artifact correction with PARAFAC, tPCA = NIRS_{ini} + artifact ($A_{1/2}$) after artifact correction with tPCA.

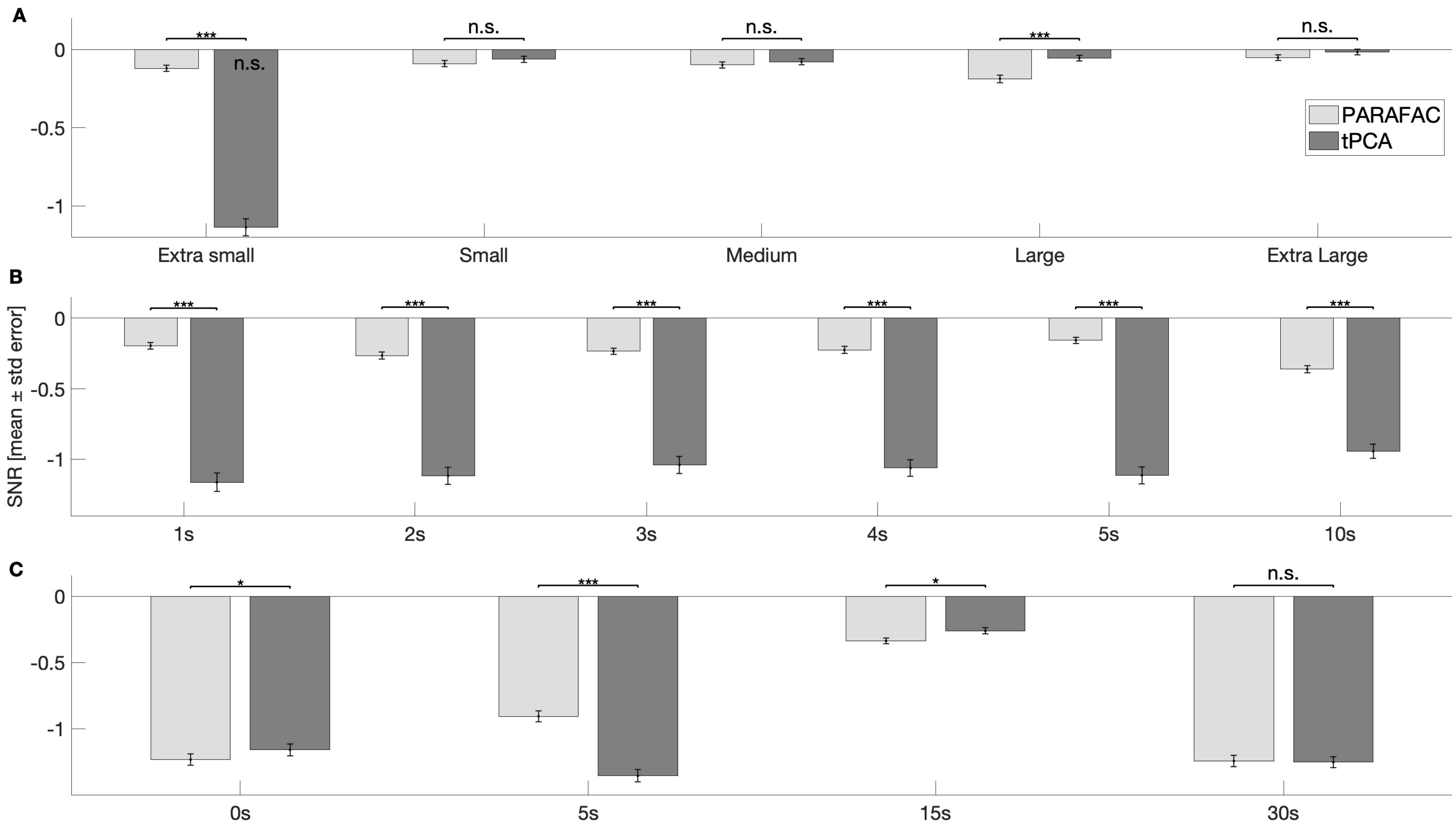

Fig. S 4 Evaluation of correction in simulated motion artifacts based on signal similarity by the use of the signal-to-noise ratio (SNR). Simulations are identified on the x-axis. A: 1a-e) artifacts with different amplitude sizes; B: 2a-f): complex artifacts with two superimposed artifacts and an onset delay between the 1st (A_1) and 2nd artifact (A_2); and C: 3a-d) the onset of the artifact relative to the beginning of a simulated HRF. An SNR closer to 0 represents a more equal ratio between the signal's variation during the artifact period and the clean signal. Results are displayed separately for the correction with PARAFAC (light grey bars) and tPCA (grey bars). The SNR of the uncorrected signal is not displayed in this figure but differed significantly from both correction techniques in all conditions, except where specified otherwise inside the bar. Significance level are based on post-hoc tests with Tukey correction. $*p \leq 0.05$, $***p \leq 0.001$, n.s. $p > 0.05$. Uncorrected = NIRS_{ini} + artifact (A_1) without correction, PARAFAC = NIRS_{ini} + artifact ($A_{1/2}$) after artifact correction with PARAFAC, tPCA = NIRS_{ini} + artifact ($A_{1/2}$) after artifact correction with tPCA.

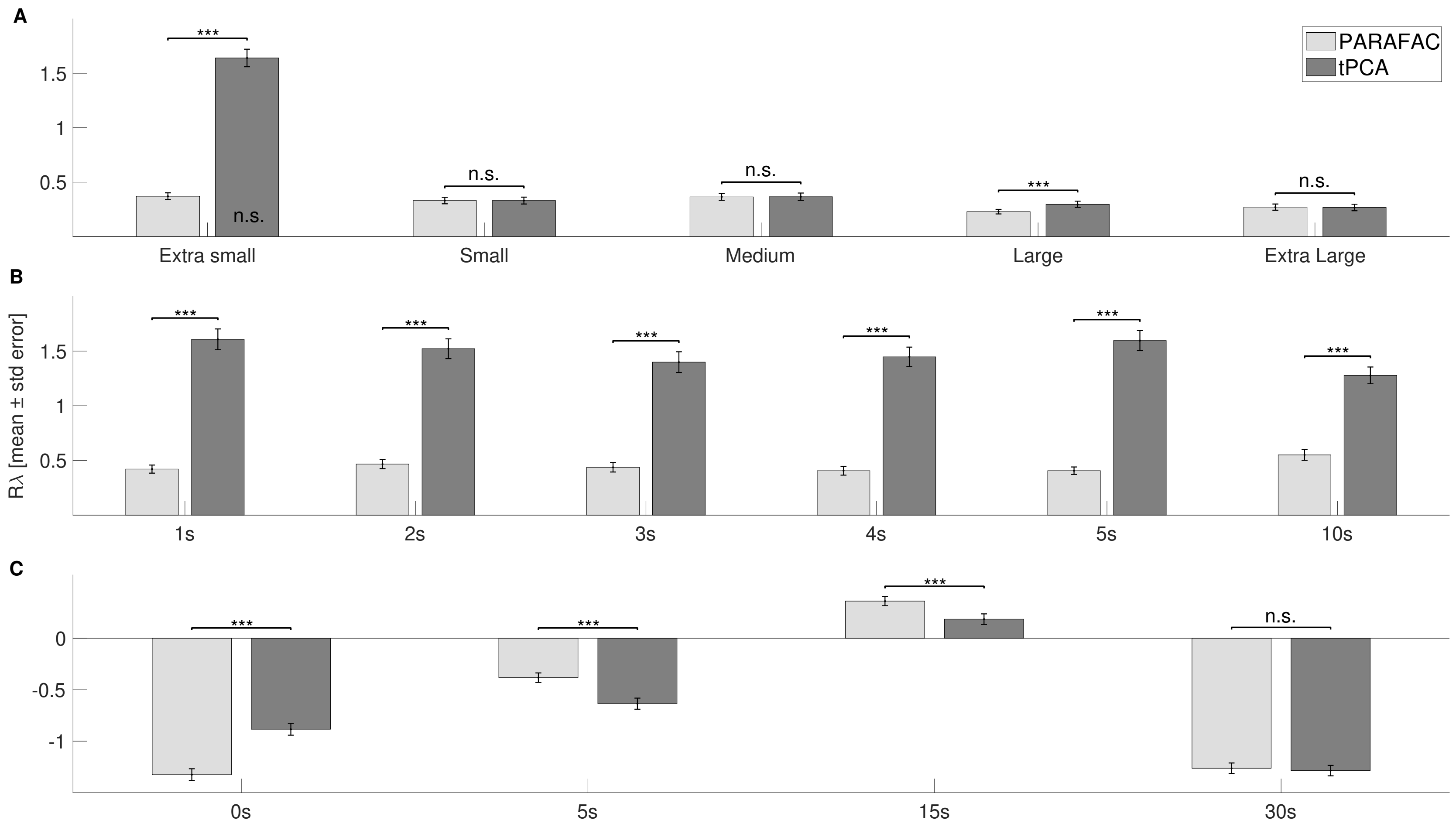

Fig. S 5 Evaluation of correction in simulated motion artifacts based on signal similarity by the use of the Pearson's correlation between wavelengths (Rλ). Simulations are identified on the x-axis. A: 1a-e) artifacts with different amplitude sizes; B: 2a-f): complex artifacts with two superimposed artifacts and an onset delay between the 1st (A_1) and 2nd artifact (A_2); and C: 3a-d) the onset of the artifact relative to the beginning of a simulated HRF. Rλ in a clean signal is usually lower than in artifacted signals. Results are displayed separately for the correction with PARAFAC (light grey bars) and tPCA (grey bars). The Rλ of the uncorrected signal is not displayed in this figure but differed significantly from both correction techniques in all conditions, except where specified otherwise inside the bar. Significance level are based on post-hoc tests with Tukey correction. $*p \leq 0.05$, $***p \leq 0.001$, n.s. $p > 0.05$. Uncorrected = NIRS$_{\text{ini}}$ + artifact (A_1) without correction, PARAFAC = NIRS$_{\text{ini}}$ + artifact ($A_{1/2}$) after artifact correction with PARAFAC, tPCA = NIRS$_{\text{ini}}$ + artifact ($A_{1/2}$) after artifact correction with tPCA.
